# Lysine-Grafted MCM-41 Silica as an Antibacterial Biomaterial

**DOI:** 10.3390/bioengineering4040080

**Published:** 2017-09-26

**Authors:** María F. Villegas, Lorena Garcia-Uriostegui, Ofelia Rodríguez, Isabel Izquierdo-Barba, Antonio J. Salinas, Guillermo Toriz, María Vallet-Regí, Ezequiel Delgado

**Affiliations:** 1 Departamento de Madera, Celulosa y Papel, Universidad de Guadalajara, Guadalajara 44100, Mexico; ib.fernanda.villegas@gmail.com (M.F.V.); gtoriz@dmcyp.cucei.udg.mx (G.T.); 2 CONACYT Research Fellow at Departamento de Madera, Celulosa y Papel, Universidad de Guadalajara, Guadalajara 44100, Mexico; lgarciaur@conacyt.mx; 3 Laboratorio de Microbiología e Inocuidad de Alimentos, Universidad de Guadalajara, Guadalajara 44100, Mexico; ofelia.rodriguez@cucei.udg.mx; 4 Departamento de Química Inorgánica y Bioinorgánica, Facultad de Farmacia, Universidad Complutense de Madrid, Instituto de Investigación Sanitaria Hospital 12 de Octubre i+12, Plaza Ramón y Cajal s/n, 28040 Madrid, Spain; ibarba@ucm.es (I.I.-B.); salinas@ucm.es (A.J.S.); 5 CIBER de Bioingeniería, Biomateriales y Nanomedicina (CIBER-BBN), 28040 Madrid, Spain

**Keywords:** lysine grafting, zwitterionization, mesoporous MCM-41 biomaterial, antibacterial adhesion, biofilm formation

## Abstract

This paper proposes a facile strategy for the zwitterionization of bioceramics that is based on the direct incorporation of l-lysine amino acid via the ε-amino group onto mesoporous MCM-41 materials. Fourier transform infrared (FTIR) studies of lysine-grafted MCM-41 (MCM-LYS) simultaneously showed bands at 3080 and 1540 cm^−1^ and bands at 1625 and 1415 cm^−1^ corresponding to -NH^3+^/COO^−^ pairs, which demonstrate the incorporation of the amino acid on the material surface keeping its zwitterionic character. Both elemental and thermogravimetric analyses showed that the amount of grafted lysine was 8 wt. % based on the bioceramic total weight. Moreover, MCM-LYS exhibited a reduction of adhesion of *S. aureus* and *E. coli* bacteria in 33% and 50%, respectively at physiological pH, as compared with pristine MCM-41. Biofilm studies onto surfaces showed that lysine functionalization elicited a reduction of the area covered by *S. aureus* biofilm from 42% to only 5% (88%). This research shows a simple and effective approach to chemically modify bioceramics using single amino acids that provides zwitterionic functionality, which is useful to develop new biomaterials that are able to resist bacterial adhesion.

## 1. Introduction

Microbial adhesion onto implanted biomaterials and the subsequent formation of biofilms is one of the major causes of failure in biomedical devices. Antimicrobial and non-fouling coatings are two strategies to prevent the attachment and spreading of microorganisms on the surface of implantable materials [1]. The use of hydrophilic or zwitterionic surfaces is among the most useful chemical strategies to avoid bacterial adhesion and biofouling [2,3,4]. In this sense, zwitterionic polymers such as poly(sulfobetaine methacrylate) (pSBMA) and poly(carboxybetaine methacrylate) (pCBMA), with mixed positively and negatively charged moieties within the same polymer chain and overall charge neutrality, exhibit ultralow fouling ability to resist non-specific protein adsorption, bacterial adhesion and biofilm formation [5,6]. 

The design of ordered mesoporous materials with resistance to bacterial adhesion is highly desirable, since these materials have unique characteristics such as high surface area and pore volume, tuneable and narrow pore size distributions, as well as ease of functionalization. These materials have been widely used for various applications, i.e., catalysis, carriers for drug storage and delivery, adsorbents [7,8,9,10], and as bioceramics for bone tissue regeneration [11,12]. In addition, ordered mesoporous silica materials have excellent properties as drug carriers, such as large loading capacity and low toxicity. Recently, there has been significant research effort on the design of bioceramics functionalized with zwitterions as a promising strategy to develop non-fouling and antibacterial adhesion surfaces [13]. These properties are related to a hydration layer on the surface, since a tightly bound water layer forms a physical and energetic barrier that prevents bacterial adhesion and non-specific protein adsorption [14,15,16,17]. It is possible to set up functionalization methods to graft both positively and negatively charged moieties onto the bioceramic surfaces. 

Thus, different zwitterionization approaches have been developed so far to prepare non-fouling bioceramics. In general, these strategies include the surface grafting with zwitterionic polymers or with low molecular moieties, involving the surface modification with either carboxylic and/or amine groups in separate steps [15,16,17,18,19]. Recently, it has been reported that the amino acids represent an alternative to provide bioceramics with a zwitterionic character. In this sense, cysteine was grafted to the surface of silica nanoparticles via a two-step procedure, showing high resistance to non-specific protein adsorption [20]. In this sense, the amino acid l-lysine (lysine) is even more attractive to create low-fouling surface owing to its low cost, high biocompatibility, and widespread availability. Lysine is a basic amino acid, which contains two primary amino groups (-NH_2_) and one carboxylic group (-COOH) with pKa for the α-amino and the carboxylic groups at 9.06 and 2.16, respectively. Consequently, at neutral pH these groups are simultaneously protonated and deprotonated. When the ε-amino group has reacted, the remaining amino group and carboxyl group form an anion-cation pair, i.e., a zwitterion, which exhibits antifouling activity [21,22,23]. For instance, Shi et al. [24] grafted lysine, glycine, and serine onto the surface of hydrolyzed polyacrylonitrile membrane with a high concentration of carboxylic acid groups. The modified membranes had similar hydrophobicity, as determined from water contact angle measurements, while the lysine-modified membrane showed the least protein fouling. Moreover, Zhi et al. [25] grafted lysine onto polydopamine-coated poly(ethylene terephthalate) that showed improved resistance to non-specific protein adsorption and platelet adhesion. 

In the present work, lysine was grafted to silica by means of cyanuric chloride (CC), which acted as a bridging molecule. CC has been shown to be an effective surface coupling agent that can react with a variety of substances, including hydroxyl and amino derivatives, and produce an ether linkage that is chemically and electrochemically stable in organic solvents and in aqueous solution (pH 3–7) [26]. CC has labile chlorine groups that can react both with the hydroxyl groups on the surface of the bioceramics and with ε-amine of lysine. Consequently, the goal of this work was to chemically modify mesoporous silica (MCM-41) using the amino acid lysine, to provide zwitterionic moieties useful for the development of new biomaterials that are able to resist bacterial adhesion and reduce the biofilm formation onto their surface.

## 2. Materials and Methods

### 2.1. Materials

l-lysine monohydrochloride (98%), tetraethyl orthosilicate (TEOS, 98%), 2,4,6-Trichloro-1,3,5-triazine (Cyanuric chloride, CC) (99%), hexadecyl trimethylammonium bromide (CTAB), and glutaraldehyde (50 wt. %) were purchased from Sigma-Aldrich (St. Louis, MI, USA). Sodium hydroxide (NaOH, 98.9%), ethylenediamine tetra acetic acid (EDTA, 98%), and copper (II) sulfate (CuSO_4_·5H_2_O, 98%) were purchased from Analytyka (Guadalajara, Mexico). Tryptic soy agar (TSA, BIOXON, Mexico City, Mexico), Tryptic soy broth (TSB, BIOXON, Mexico City, Mexico) and Todd Hewitt Broth (THB, Fluka analytical, Toluca, Mexico) were used as received. The microorganisms *Escherichia coli* (ATCC 25922), and *Staphylococcus aureus* (ATCC 29213) were obtained from the American Type Culture Collection (Manassas, VA, USA). All the other reagents were analytical grade and used without further purification.

### 2.2. Preparation of MCM-41 with Zwitterionic Moieties

MCM-41 silica was synthesized by the sol-gel method according to Cai et al. [27]. In brief, CTAB (1 g) was dissolved in 480 mL of distilled water, and then 3.5 mL of NaOH (2 M) were added, the temperature raised to 85 °C and stirred for 30 min. TEOS (5.0 mL) was then introduced drop-wise to the surfactant solution. Once the addition of TEOS was completed, the mixture was stirred for 2 h at 85 °C. The white precipitates were filtered, washed with ethanol, dried under vacuum at room temperature for 48 h, and calcined in air at 550 °C for 4 h with a heating rate of 1 °C/min.

The resulting bioceramic was functionalized with the amino acid lysine (MCM-LYS) to confer its zwitterionic character. The modification of MCM-41 with the amino acid lysine was carried out following the procedure reported by Delgado et al. [28]. In brief, l-lysine hydrochloride was first converted into the copper complex as it follows: lysine (2.32 g in 12.72 mL NaOH 1 M) and copper sulfate (1.59 g CuSO_4_·5H_2_O in 30 mL H_2_O) were brought together and stirred into a homogeneous solution. The pH of the solution was adjusted to 7.0 with NaOH (1 M) and cooled to 0 °C. MCM-41 was grafted with CC as follows: 1 g MCM-41 was placed in a water–acetone mixture (80 mL, 75/25) under constant stirring and cooled at 0 °C. Afterwards, the CC (2.34 g) solution in p-dioxane (40 mL) was added under magnetic stirring. Subsequently, NaCl (1.5 g) was added in two equivalent portions, and the pH of the mixture was adjusted to 12 with NaOH (6 M) and kept at 0 °C for 30 min under constant stirring. The coupling of lysine to the grafted MCM-41 was achieved by immediately adding the lysine-copper complex under vigorous stirring and leaving it to react for 15 min. Then, the reaction mixture was removed from the ice bath and placed in a water bath at room temperature, followed by a slow increase of temperature until it reached 65 °C, and then kept stable for 40 min. The functionalized bioceramic was filtered off and washed with water and p-dioxane several times to remove CC residues. The copper was completely eliminated from the lysine copper complex by suspending the functionalized bioceramic in 40 mL distilled water with 1.18 g EDTA (twice, the second time adding 0.1% triton X-100 detergent), boiled at 100 °C for 10 min, filtered, treated with 0.05 M acetic acid (30 mL) and extensively washed with water and dried overnight under vacuum at 60 °C.

### 2.3. Characterization

The structural characteristics of the resulting materials were determined by powder X-ray diffraction (XRD) in a Siemens D500 diffractometer (Siemens, Eindhoven, The Netherlands) equipped with Cu Kα (40 kV, 20 mA) over the range from 2° to 10° with a step of 0.005 and a contact time of 4 s. The nitrogen adsorption isotherms were measured at −196 °C with a Micromeritics ASAP 2020 analyzer (Micromeritics, Norcross, GA, USA). In all cases, the samples were degassed at 60 °C for 24 h before analysis. Electron microscopy was carried out in a JEOL JEM-220FS transmission electron microscope (JEOL, Tokyo, Japan) operating at 300 kV. The chemical composition and the presence of functional groups on the synthesized materials was determined using Fourier transform infrared (FTIR) spectroscopy in a Thermo Nicolet Nexus spectrometer equipped with a Goldengate attenuated total reflectance (ATR) device (Thermo scientific, Waltham, MA, USA) from 4000 to 400 cm^−1^. Quantitative determination of chemical composition of the samples was carried out by elemental chemical analysis in a LECO CHNS-932 microanalyzer (Saint Joseph, MI, USA). Thermogravimetric analyses (TGA) were carried out in nitrogen between 30 and 600 °C (flow rate of 50 mL/min, heating rate of 10 °C/min) using a Perkin Elmer TGA Diamond analyzer (PerkinElmer, Akron, OH, USA). Zeta-potential (ζ) measurements were performed on a Malvern Zetasizer Nano Series instrument (Malvern Instruments Ltd., Malvern, UK) with 55 mg of each sample in 30 mL of 10 mM KCl (used as the supporting electrolyte), the mixture was vigorously stirred to reach a homogenous suspension and the pH adjusted by adding appropriate volumes of 0.1 M HCl or 0.1 M KOH solutions.

### 2.4. Bacterial Adhesion Assays

Bacterial adhesion assays on MCM-41 and MCM-LYS were tested using gram negative *E. coli* (assay A) and gram positive *S. aureus* (assay B) by using an established methodology [29,30,31,32,33]. In brief, disk-shaped pieces of 6 mm diameter and 1 mm height were prepared by compacting fractions of 30 mg of dried powders using 2.75 and 3 MPa uniaxial and isostatic pressure, respectively. Before the adhesion assay, samples were sterilized by UV irradiation for 10 min on each side of the piece and then stabilized in sterile phosphate-buffered saline (PBS) for 2 h. 

Assay A: *S. aureus* strain was grown to a mid-logarithmic phase in THB medium at 37 °C under orbital stirring at 100 rpm until the optical density measured at 600 nm reached 0.5 in an UV/VIS spectrometer (UV-530, Bonsai technologies, Madrid, Spain). Bacteria from the culture were collected by centrifugation (Labofuge 400 centrifuge, Thermo Scientific, Waltham, MA, USA) at 1500 rpm for 10 min at room temperature, washed three times with sterile PBS (pH 7.4), and re-suspended in 12 mL of PBS. In this study (Assay A), PBS solutions with pH 3.6 and 7.8 were also used. 

Assay B: *E. coli* was grown in TSB at 37 °C under orbital stirring at 100 rpm until the optimal density, as measured at 600 nm, reached 0.5. At this point, bacteria from the culture were collected by centrifugation at 1500 rpm for 10 min at room temperature, washed three times with sterile PBS (pH 7.4), and subsequently re-suspended in 12 mL of sterile PBS.

Then, different disk-shaped samples were soaked in 1 mL of each bacterial suspension (A or B) and incubated at 37 °C under orbital stirring at 100 rpm for 90 min.

The quantification of bacteria attached to the biomaterial surfaces after the bacterial adhesion assays (A and B) was performed by a method described elsewhere [29,30]. The bioceramic samples were aseptically removed and rinsed three times with sterile PBS to eliminate any free bacteria [32]. Each disk was placed in 1 mL sterile PBS in Eppendorf vials (Nirco, Madrid, Spain), followed by 10 min sonication in a low-power bath sonicator (Selecta, Madrid, Spain). This sonication process was carried out three times, assuming then that 99.9% of adhered bacteria were removed. Thereafter, 100 µL of each sonication product were cultivated on TSA plates, followed by incubation at 37 °C overnight. Determination of the number of colony forming units (CFU) resulting from the overall sum of the three sonication stages allowed the determination of the number of bacteria initially adhered onto the disks. The experiments were performed in duplicate. Surface characterization of the samples after 90 min in *E. coli* bacterial incubation was performed by SEM in a JEOL model JSM-6335F microscope (JEOL, Tokyo, Japan). Before the SEM studies, the attached bacteria were fixed with 2.5 vol. % glutaraldehyde in PBS, pH 7.4 and dehydrated by slow water replacement using a series of graded ethanol solutions (10%, 30%, 50%, 70% and 100%) in deionized water, with a final dehydration step in absolute ethanol before critical point drying (Baltec AG, Balzers, Liechtenstein) [33]. The materials were mounted on stubs and gold plated in a vacuum using a sputter coater (Baltec AG, Balzers, Liechtenstein) and visualized by SEM.

### 2.5. S. aureus Bacterial Biofilm Formation

Biofilm growth onto MCM-41 and MCM-LYS surfaces was determined. Briefly, *S. aureus* biofilms were developed by suspending the disks of each material in a bacteria solution of 10^8^ bacteria per mL during 48 h at 37 °C and orbital stirring at 100 rpm. In this case, the medium used was 66% tryptic soy broth TSB (BioMerieux, Marcy L’Etoile, France) +0.2% glucose to promote robust biofilm formation. After 90 min of incubation, the disks were washed three times with sterile PBS, stained with a 3 μL/mL of Live/Dead^®^ Bacterial Viability Kit BaclightTM (Thermo Fisher Scientific, Waltham, MA, USA) and 5 μL/mL of Calcofluor solution to specifically determine the biofilm formation, staining the mucopolysaccharides of the biofilm (extracellular matrix in blue). Both reactants were incubated for 15 min at room temperature. Biofilm formation was examined in an Olympus FV1200 confocal microscope (Olympus, Tokyo, Japan), by taking eight photographs of each sample (60× magnification) [34]. The surface area covered with adhered bacteria was calculated using ImageJ software (National Institutes of Health, Bethesda, MD, USA). The experiments were performed in triplicate, and the results were expressed as the mean ± standard deviation.

## 3. Results

**Figure 1:**
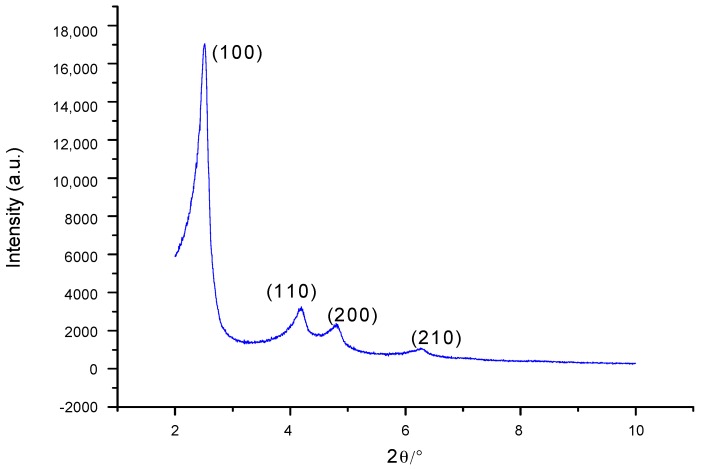
XRD patterns of pristine mesoporous silica (MCM-41). Characteristic diffraction patterns of ordered mesoporous silica corresponding to 2D hexagonal structure, p6mm plain group is observed.

**Figure 2:**
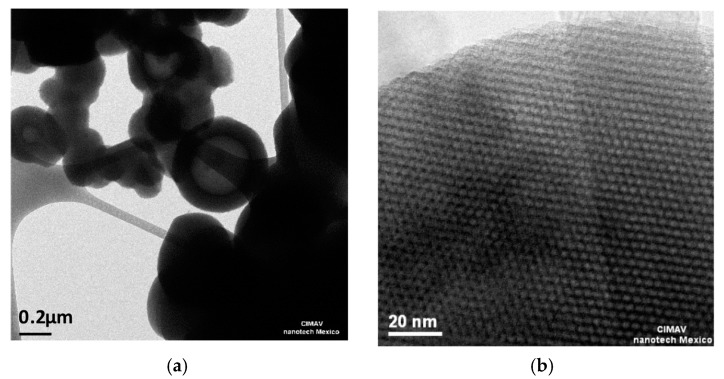
Transmission electron microscope (TEM) images of MCM-41: (**a**) The image shows aggregates of circular particles between 200–500 nm; (**b**) The image shows the surface of a particle demonstrating a honeycomb array with a regular pore size of about 2 nm.

MCM-41 was successfully synthesized by the sol-gel methodology, as shown in the following analyses. In Figure 1, the XRD pattern exhibits a strong (100) reflection peak with three small peaks (110), (200), and (210) typical of MCM-41 material [35]; the formation of MCM-41 particles with diameters of around 0.2 µm with the ordered 2D hexagonal array and straight structural features of MCM-41 are clearly revealed by TEM, as observed in Figure 2a,b. It is worth noticing that the synthesized MCM-41 did not present intergrowth or intertwinned aggregations. 

**Scheme 1:**
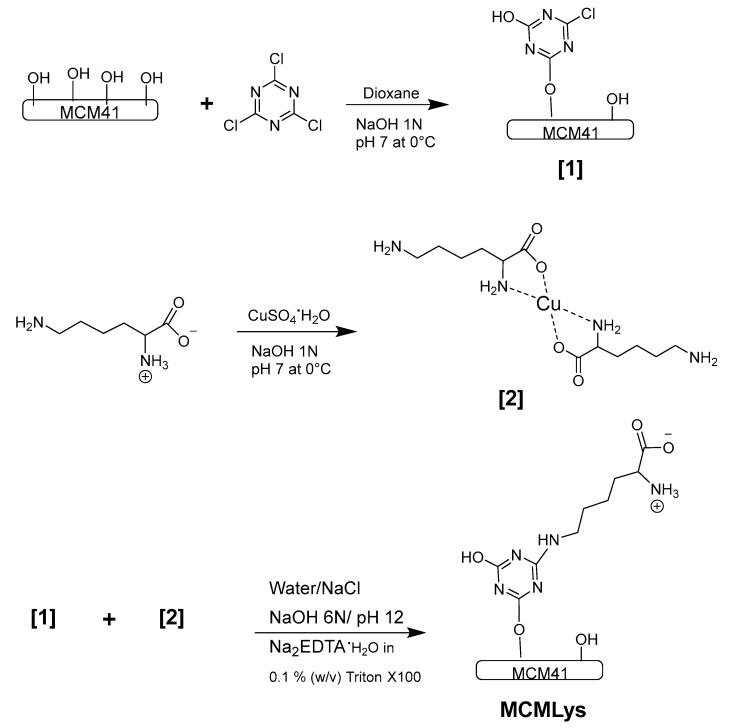
Lysine grafting via coupling with cyanuric chloride (CC) onto MCM-41. Silanol groups on MCM-41 first react with CC, which in turn react with the ε-amino group of a Cu^2+^ lysine complex. After treatment of the product of [1] and [2] with EDTA to remove copper, the proper zwitterionic function on the bioceramic is achieved.

Lysine was grafted onto MCM-41 silica using CC as linking agent, as shown in Scheme 1. The surface hydroxyl groups of MCM-41 react with chlorine atoms of CC in a first stage. The remaining chlorine atoms in CC can react with the ε-amino group of lysine (Compound **1**) in a second stage. The protection of α-amino and carboxyl groups is achieved by complexing them with Cu^2+^ (Compound **2**); the free ε-amino groups of the copper complex reacted with Compound **1** to obtain the MCM-LYS material. 

**Figure 3:**
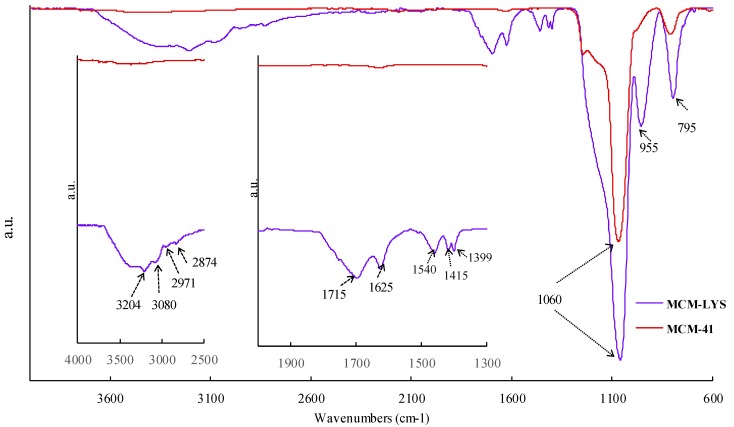
Fourier transform infrared (FTIR) spectra of MCM-41 and lysine-grafted MCM-41 (MCM-LYS). The insets show regions from 4000–2500 cm^−1^ (**left**) and 2000–1300 cm^−1^ (**right**) to better appreciate the changes arising from the grafting of pristine MCM-41.

Figure 3 shows the FTIR spectra of pristine MCM-41 and MCM-LYS. MCM-41 shows the characteristic band of the siloxane group (-Si-O-Si-) at 1060 cm^−1^, whereas the bands at 955 and 795 cm^−1^ correspond to -Si-OH and -Si-O, respectively [14]. The new bands, shown in the inset, at 3204 and 1399 cm^−1^, originate from N-H stretching and deformation frequencies, respectively. Furthermore, the presence of zwitterionic pairs can be demonstrated by observing the peaks at 3080 and 1540 cm^−1^, which correspond to -NH^3+^ stretching and deformation frequencies, respectively, and the bands at 1625 and 1415 cm^−1^, which are typical of the antisymmetric and symmetric frequencies of ionic carboxyl (COO^−^). The carbonyl group of lysine is observed in 1715 cm^−1^, while the bands observed in 2971 and 2874 cm^−1^ correspond to -CH_2_- of the amino acid chain. It was therefore established that MCM-41 was successfully modified with lysine, and that the samples exhibit a zwitterion moieties due to the presence of NH^3+^ and COO^−^ groups (see insets in Figure 3).

**Figure 4:**
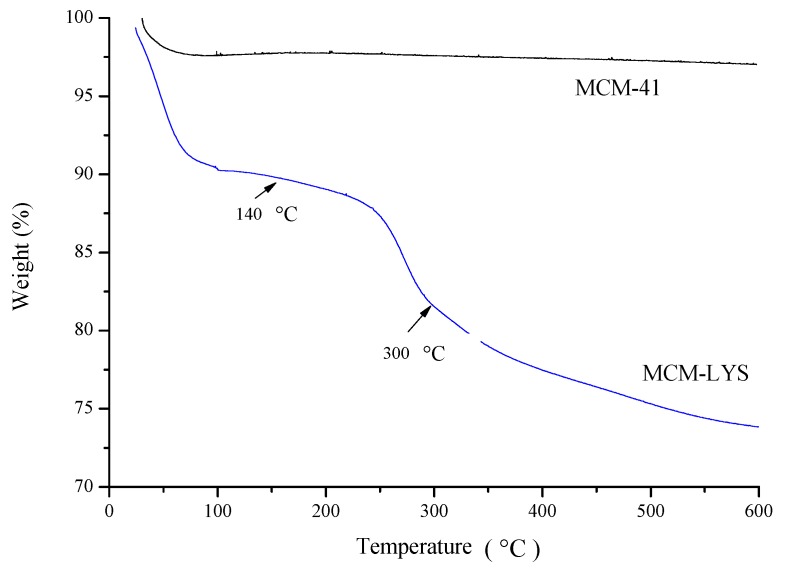
Thermogravimetric analyses (TGA) curves of MCM-41 and MCM-LYS. The thermogram of MCM-LYS shows weight loss in three different regions: 25–140 °C, 140–300 °C, and 300–600 °C.

The textural properties and the organic elemental analysis of MCM-41 and MCM-LYS are summarized in Table 1. It can be seen that the surface area and pore volume of MCM-LYS considerably decreased to 62% and 55%, respectively, in comparison with pristine MCM-41. This reduction of surface area and pore volume is due to the blocking of pores as mesoporous silica is being functionalized. Furthermore, the organic elemental analysis showed that MCM-LYS had 2.47, 2.53, and 5.95 wt. % N, H, and C content, respectively, whereas pristine MCM-41 showed no organic elements (except for hydrogen) in its composition. These facts indicate that lysine was successfully attached onto the bioceramic. The amount of lysine attached to the bioceramic was calculated by taking into account the amount of CC that was previously attached for coupling. In parallel, TGA of MCM-LYS and MCM-41 was carried out up to 600 °C. It was found that the TGA curve of MCM-LYS, which is shown in Figure 4, had two step changes: one at 100–140 °C, which corresponding to the loss of water molecules entrapped in the bioceramic, and the other at 200–300 °C from lysine decomposition. Then, the curve drifted, showing the decomposition of the remaining organic matter that must correspond to CC, which is attached to the bioceramic. From these transitions, it was found that lysine was attached in about 8 wt. %, and that CC was attached in about 7 wt. %, with a decrease in organic material for about 15 wt. %. These numbers are in agreement with the result of the elemental analysis presented in Table 1, which showed an organic content of about 11%.

It is noteworthy that MCM-LYS retained up to 10% of the weight of water, as opposed to bare MCM-41, which indicates that the grafted zwitterions were capable to form a layer of water on the surface of the material that in turn might inhibit the adhesion of bacteria [4].

**Figure 5:**
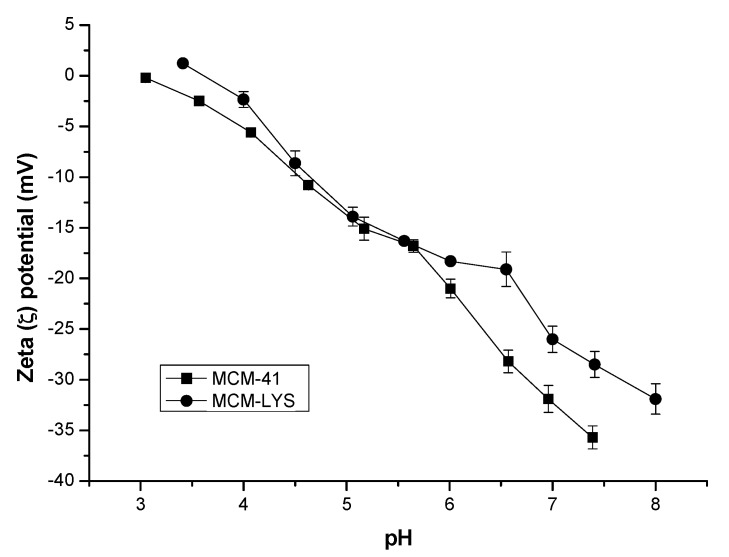
ζ-Potential vs. pH of MCM 41 and MCM-LYS.

ζ-potential was measured to determine the isoelectric point (IEP) and the electrostatic behavior of MCM-41 and modified MCM-41 at physiological pH in PBS (pH = 7.4). Figure 5 shows that MCM-41 has a near zero charge at pH 3, whereas MCM-LYS has an IEP at pH 3.6. At physiological pH 7.4, MCM-41 and MCM-LYS show a ζ-potential of −35 and −28 mV, respectively. In the case of MCM-41, this is due to ionized silanol groups (-Si-O-), whereas in the case of MCM-LYS, it is owed to the presence of -Si-O- groups unreacted in the grafting process, since lysine is in its zwitterionic form. Similar behavior was observed by Shi et al. [24], when lysine was grafted onto polyacrylonitrile membrane.

**Figure 6:**
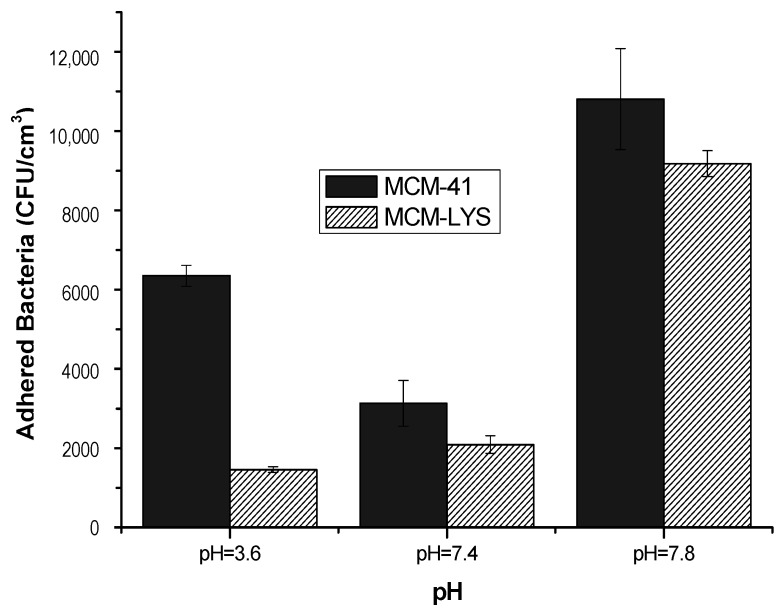
*S. aureus* CFU/cm^3^ after 90 min cultivation in the presence of MCM-41 and MCM-LYS at the studied pHs.

To evaluate the *S. aureus* adhesion on the bioceramics, *in vitro* assays were carried out at 37 °C for 90 min at different pH levels (assay A). Figure 6 shows the CFU adhered to the different bioceramics as a function of pH. It can be seen that MCM-LYS had the lowest bacterial adhesion regardless of pH, showing a decrease of about of 77%, 33%, and 15% as compared with pristine MCM-41, at pH of 3.6, 7.4, and 7.8 respectively. It is noteworthy that at pH 3.6, MCM-LYS showed the lowest bacterial adhesion, which corresponds to its isoelectric point, i.e., the pH at which the electric charges are neutralized.

**Figure 7:**
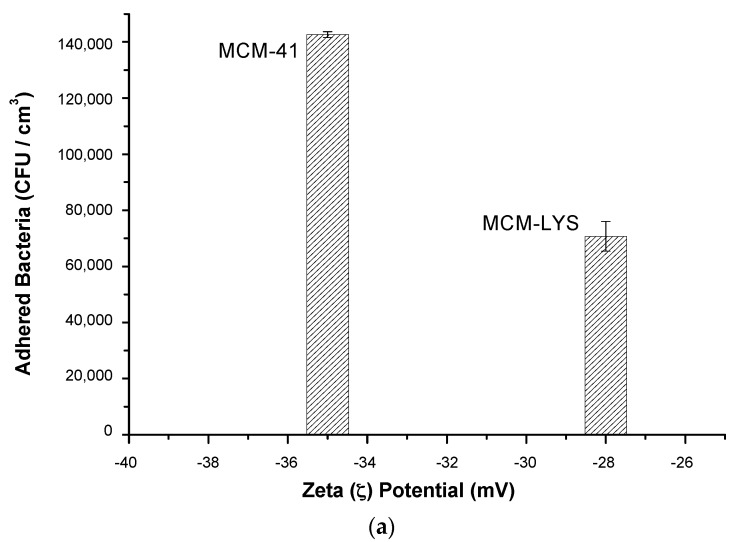

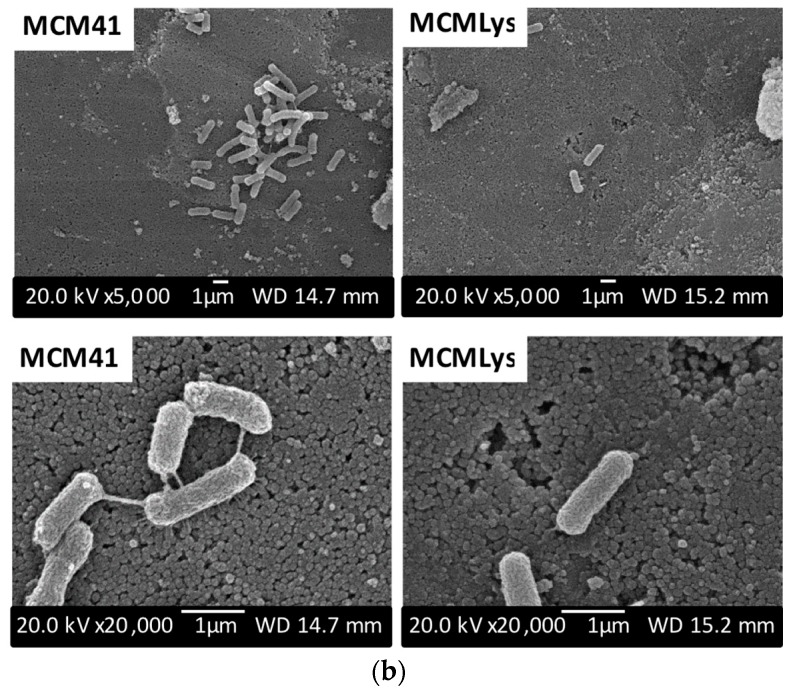
(**a**) *E. coli* colony forming units (CFU)/cm^3^ after 90 min cultivation in the presence of MCM-41 and MCM-LYS at physiological pH (7.4); (**b**) SEM micrographs at 5000× and 20,000× of MCM-41 and MCM-LYS after 90 min cultivation of *E. coli*.

Studies of *E. coli* adhesion onto the samples were carried out only at the physiological pH of 7.4. Figure 7a shows that MCM-LYS has the lowest bacterial adhesion, which decreased 50% as compared with pristine MCM-41. Moreover, Figure 7b depicts SEM images of the adhesion of *E. coli* on the surface of both materials, which confirm the results obtained by counting the CFU. SEM micrographs of a MCM-LYS sample show a lower amount of *E. coli* bacteria as compared with MCM-41. In addition, the SEM images show that bacteria are interconnected in MCM-41. In contrast, in MCM-LYS bioceramic, the amount of bacteria observed is smaller, and they appear isolated without apparent communication between them. These results show that zwitterionic moieties on the surface of MCM-LYS, provided by lysine, decreased the initial attachment of both bacteria [31]. In absolute terms, the adhesion of *E. coli* was higher on MCM materials, as compared with *S. aureus*; however, the relative effectiveness of the zwitterionic pair was more pronounced on *E. coli*, which was shown as a reduction of 50% adhesion on MCM-LYS.

**Figure 8:**
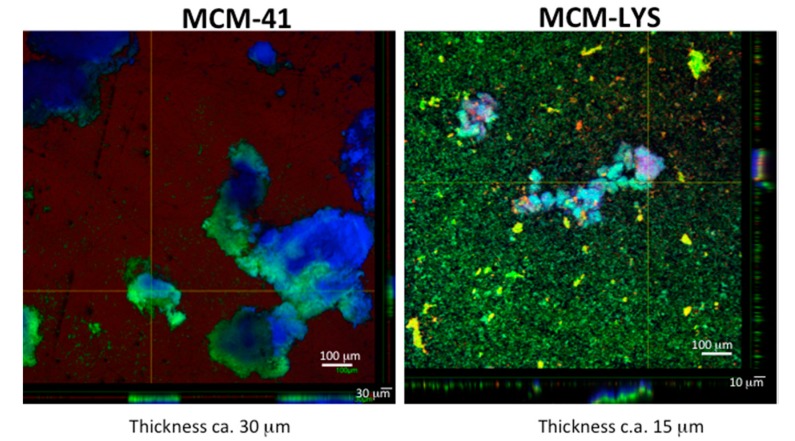
*S. aureus* biofilm grown on MCM-41 and MCM-LYS samples, respectively; the images were collected by confocal microscopy after 48 h of incubation (the larger scale bars correspond to the area of the figure, whereas the smaller scale bar refers to the thickness).

Confocal microscopy was carried out to characterize the biofilm formation onto MCM-LYS and bare MCM-41 after 48 h of incubation using Syto-9/propidium iodide dyes (to stain alive and dead bacteria in green and red, respectively), and Calcofluor fluorescent stains (to stain the extracellular matrix of biofilms in blue). The thickness of the biological material attached to both surfaces was determined by analyzing eight different areas of each piece by confocal microscopy. The measured thickness was 30.3 ± 3.3 µm for the bare MCM-41 substrate (*n* = 8 and *p* < 0.001, Kruskal-Wallis test), while the measured thickness was 15.3 ± 7.8 µm for MCM-LYS. Moreover, Figure 8 depicts a compact biofilm layer formed by large colonies with a size >500 µm for MCM-41. However, for the MCM-LYS sample, the scenario is completely different, showing smaller colonies (<50 µm). These results reveal the limited ability of *S. aureus* to form biofilm onto a lysine-functionalized surface as compared with bare MCM-41. 

## 4. Discussion

Different zwitterionization approaches have been developed to prepare non-fouling bioceramics, which include surface grafting with zwitterionic polymers, separately grafting low molecular moieties containing carboxylic and/or amine groups, or with cysteine [15,16,17,18,19,20]. In this contribution, a facile strategy for creating a non-fouling, antibacterial bioceramic surface is reported. MCM-41 bioceramic was synthesized, and thereafter functionalized with lysine in order to create surface zwitterionic pairs. The use of cyanuric chloride as a coupling agent between MCM-41 and the amino acid warrants mild reaction conditions, and also acts as a bridge to effectively connect the copper complexed l-lysine through its ε-amino group. A battery of analytical techniques was used to confirm that l-lysine was covalently attached to the surface of the bioceramic while maintaining its zwitterionic character. For example, ATR–FTIR analysis established that the zwitterionic pair was present, as demonstrated by the simultaneous appearance of bands at 3080, 1540 cm^−1^ (NH^3+^), and at 1625 and 1415 cm^−1^ (COO^−^). The pristine bioceramic had a large surface area and pore volume, which after functionalization were significantly reduced due to pore blockage. Organic elemental analysis corroborated the presence of organic elements such as C, H, N, whereas neat MCM-41 contained only hydrogen; the approximate amount of grafted lysine was 8 wt. %, which was confirmed both with TGA and elemental analysis. TGA revealed a very important fact: the amount of water retained by the modified bioceramic was 10 wt. %, whereas bare MCM-41 only retained about 2 wt. %. This fact indicates that the grafted zwitterions allow the surface to retain five-fold more water as compared with bare MCM-41. This layer of water elicits a resistance to bacterial adhesion [36,37], which is critical for biofilm formation. It has been shown that a single compact layer of mixed charged groups, such as zwitterions, play a dominant role in surface resistance to non-specific protein adsorption due to strong binding of water molecules, which form a physical and energetic barrier that prevents bacterial adhesion [14,15,16,17,38]. ζ-potential revealed that both surfaces were negatively charged at physiological pH, due to ionized silanol groups, and that the IEP was slightly increased for the modified bioceramic. MCM-LYS showed the lowest adhesion of *S. aureus,* especially at the IEP. At physiological pH, MCM-LYS showed even lower adhesion for *E. coli* (50%) as compared with unmodified MCM-41. These results showed that the zwitterionic moieties on the modified bioceramic, provided by lysine, decreased the initial attachment of both bacteria [31]. Confocal microscopy images confirmed the limited ability of *S. aureus* to form a biofilm (88% less) in the lysine-modified bioceramic. The facile method here described for incorporation of zwitterionic functions onto bioceramics opens up a great opportunity to develop new biomaterials resistant to bacterial adhesion.

## Figures and Tables

**Table 1:** Textural data and elemental analysis of MCM-41 and MCM-LYS.

Sample	S_BET_ (m^2^/g)	Vp (cm^3^/g)	Dp (nm)	Elemental Analysis			% CC/Lys
				C (%)	H (%)	N (%)	
MCM-41	954	0.95	4.96	----	0.79	----	----
MCM-Lys	359	0.43	4.80	5.95	2.53	2.47	11
